# Preparation of an Active Dressing by In Situ Biosynthesis of a Bacterial Cellulose–Graphene Oxide Composite

**DOI:** 10.3390/polym14142864

**Published:** 2022-07-14

**Authors:** Tobiasz Gabryś, Beata Fryczkowska, Janusz Fabia, Dorota Biniaś

**Affiliations:** 1 Department of Material Science, Faculty of Materials, Civil and Environmental Engineering, University of Bielsko-Biala, Willowa 2, 43-309 Bielsko-Biala, Poland; jfabia@ath.bielsko.pl; 2 Department of Environmental Protection and Engineering, Faculty of Materials, Civil and Environmental Engineering, University of Bielsko-Biala, Willowa 2, 43-309 Bielsko-Biala, Poland; bfryczkowska@ath.bielsko.pl (B.F.); dbinias@ath.bielsko.pl (D.B.)

**Keywords:** bacterial cellulose, graphene oxide, biosynthesis, composite membranes, nanofibers

## Abstract

This paper presents a simple method of obtaining a bacterial cellulose (BC) composite with the addition of graphene oxide (GO) using an in situ method and studies the influence of GO nanoparticles on the structure and properties of the obtained membranes. Microorganisms obtained from Golden Delicious apple vinegar were used to obtain the BC. During the biosynthesis, GO was introduced in the amounts of 3.7, 5.4 and 7.1% *w*/*w*. The resulting BC/GO composite was characterized by high water content (~400%), a thickness of about 1.1 mm (in wet form) and a cellulose nanofiber diameter of ~100 nm. The possibility of using the resulting composite membranes as potential active dressings with the sustained-release analgesic medicine—paracetamol—was investigated. The BC/GO composite membranes were characterized by a medicine sorption of 60 mg/g of BC, a slow desorption time, a constant medicine concentration over time and an 80% paracetamol release rate after 24 h. The morphology of membrane surfaces and cross-sections were examined by means of scanning electron microscopy (SEM). Infrared spectroscopy (FTIR), X-ray structure studies (WAXS) as well as thermal analysis (TGA) demonstrated the presence of GO in the BC matrix and interactions between the matrix and the additive.

## 1. Introduction

Cellulose is an inexpensive and readily available biopolymer synthesized by plants. It is obtained by the processing of coniferous and deciduous trees, as well as by recycling used paper. Plant-based cellulose occurs together with supporting substances such as lignin, pectin and hemicellulose. Such a chemical composition does not affect the wide possibilities of application of this biopolymer. However, when used as a biomaterial, 100% cellulose content products are expected. For this purpose, BC can be employed.

BC can be synthesized by different bacteria, including Gram-negative bacteria, such as *Gluconacetobacter xylinus* [1,2], *Agrobacterium* [3] and *Rhizobium* [4], and Gram-positive bacteria, such as *Sarcina* [5]. As many as 59 strains of bacteria from ripe fruit and vegetables that can synthesize BC have already been isolated and tested [5].

Cellulose can also be produced in vitro, i.e., using the cell-free enzyme system. This method employs enzymes derived from bacteria or fungi [6,7,8,9]. The cell-free system method has many advantages over bacteria-based methods. It uses less glucose, and the efficiency of the process is higher than that obtained with bacteria. The resulting fibers are characterized by larger diameters than in the method based on the use of microorganisms [9]. Cellulose produced by means of cell-free enzyme systems is characterized by better physicochemical and structural properties [10]. BC is synthesized in the bacterial cell membrane from nucleotide-activated glucose [11]. Bacteria synthesize BC through the pores of the cell membrane in the form of fibrils composed of D-glucose units, which are connected by β-1,4-glycosidic bonds. Bacteria synthesize cellulose nanofibers through 50 to 80 channels located on the surface of the bacterial cell membrane [10]. In the first stage of synthesis, glucan chains form protofibrils, which combine into nanofibrils to form a compact, ultrathin three-dimensional network [11]. The resulting BC fibers have a diameter of up to 100 nm and a length of 10 μm [12]. The production rate of BC is approximately 200,000 glucose units per second [13]. Single BC fibers are 100 times thinner than those of plant origin, and the nano-nonwoven fabric produced during synthesis is characterized by a high water retention index [10]. The BC synthesis mechanism influences some specific properties of the material, such as: specific surface area (high aspect ratio of fibers with diameter 20–100 nm), porosity, mechanical strength (Young’s modulus 15–18 GPa), degree of crystallinity (up to 80%) and polymerization, high water-holding capacity (over 100 times of its own weight) or biodegradability [5,12]. As a hydrogel, BC has the ability to absorb, store and desorb large amounts of water, which is possibly due to its nanofiber structure [14]. This makes BC an excellent biomedical material, along with biopolymers such as chitosan, alginates, or hyaluronic acid and collagen [15]. BC, combined with those biopolymers, forms composite materials. The literature describes the possibility of synthesizing BC/chitosan composite as a dressing material for the treatment of various types of wounds, burns and ulcers [16] or as a tissue culture material (scaffold) [17]. BC/alginate composites are used in tissue engineering [18]. Additionally, the combination of three biopolymers, BC/alginate/chitosan, makes it possible to obtain an antibacterial dressing [19]. BC can also be combined with hyaluronic acid [20] and collagen [21,22], and the resulting composites can be used in bone regeneration.

BC is also potentially useful in 3D printing [23,24]. The literature provides a method of printing with an ink containing acetic bacteria, which produces BC directly in the resulting printout, creating a tissue scaffold [25]. To print BC, composites with alginates are formed and scaffolds for cartilage culture are obtained by means of an electromagnetic jet printer [26]. This polymer can be chemically modified to obtain a three-dimensional material that has potential for application in soft tissue engineering [27].

There are several methods of modifying BC in order to give it new, previously undescribed properties. Two known methods of BC modification are in situ and ex situ [4]. The ex situ modification is relatively simple and consists of introducing additives or nano-additives to the finished BC product. Barud et al. obtained a non-genotoxic and non-cytotoxic BC/silk fibroin composite [28]. Other researchers have modified BC by introducing polyethylene glycol, silver nanoparticles or zinc oxide [4]. The literature also mentions the possibility of chemical modification of BC by reaction of hydroxyl groups of cellulose with gelatin [29], hydroxyapatite [4], graphene oxide [30] and proteins [12].

The second method of BC modification is the in situ method, which consists of introducing additives or nanoparticles that are soluble in water to form dispersion with it, directly into the culture medium. There are many scientific reports of BC modified with the use of electrically conductive polymers [31], carbon nanoadditives, including graphene [32] or graphene oxide [33,34]. The use of carbon nanoadditives increases the specific surface area of the composite, increasing its porosity, improving its strength properties and giving it electrically conductive properties [32].

The third method of BC modification is solvent dissolution–regeneration. This method consists of dissolving BC, then introducing the modifier, followed by BC regeneration. Jayani et al. obtained probiotic carriers by dissolving BC in trifluoroacetic acid, mixing with polyvinyl alcohol (PVA) solution, and then producing nanofibers via electrospinning [35].

Graphene oxide (GO) is a particularly interesting carbon nanomaterial that can be used to produce a BC composite. Known as a two-dimensional nanomaterial with many oxygen functional groups on its surface, graphene oxide links strongly to BC, forming hydrogen bonds [36]. The literature reports on many types of BC/GO nanocomposites with different conformations, developed for absorbents for the elimination of pollutants. Luo et al. reported a method of preparing ultra-strong BC/GO nanocomposites by multiple in situ layer-by-layer assembly steps which consisted of spraying GO dispersed medium onto a growing static BC membrane [36]. In addition, graphene nanosheets have been incorporated into BC growing membranes by spraying to obtain flexible and highly conductive nanopapers for applications in energy storage devices [32,37]. In the area of biomedicine, these carbon-based materials have been used for the development of biosensors, in tissue engineering and as delivery carriers for genes or medicines [38]. To date, none of the GO applications have been approved for clinical trials due to issues related to potential toxicity in model animals, and there are several recent studies in which the in vitro biocompatibility and antimicrobial activity of nanocomposites containing GO have been assessed [39,40]. BC/GO hydrogels have been developed in static and dynamic cultivation to develop systems for the release of medicines such as doxorubicin hydrochloride or ibuprofen, although low release profiles have been obtained [30,36,41].

The experiment describes a simple method of obtaining a composite of BC with the addition of graphene oxide (in the amounts of 3.7, 5.4 and 7.1% *w*/*w*) using the in situ method. In this study, microorganisms obtained from Golden Delicious apple vinegar, rather than selected bacterial strains, were used. No additional nutrients for bacterial growth were added. Additions such as peptone, yeast extract, citric acid and Na_2_HPO_4_ were not used in the BC for synthesis [14]. Instead, a simple method is proposed for synthesizing the BC/GO composite with the following properties: (~400%) and wet thickness (~1100 µm), as well as cellulose nanofiber diameter of ~100 nm. The physicochemical properties of BC/GO could be used as a potential active dressing with prolonged analgesic effect. BC/GO composite membranes are characterized by paracetamol sorption at the level of 60 mg/g of BC and a slow desorption time, releasing approximately 80% of the medicine after 24 h. Structural tests (WAXS, FTIR, TGA) confirm the presence of GO in membranes and its influence on physicochemical properties.

## 2. Materials and Methods

### 2.1. Chemicals

The reagents for the preparation of liquid medium and purification of obtained BC included saccharose (≥99%) and potassium hydroxide (≥97%) purchased from Sigma-Aldrich.

The reagents for the production of graphene oxide included: graphite powder < 20 μm purchased from Sigma-Aldrich and potassium permanganate (≥99%), sulfuric acid (98%) and hydrogen peroxide (30%) purchased from Avantor Performance Materials Poland S.A (Gliwice, Poland). Graphene oxide (GO) was obtained using the modified Hummers method [42]. GO synthesis description and testing of its properties (XRD, DSC, FTIR) were very similar to those of our earlier work [43]. After the synthesis was completed, the concentration of GO in wet graphene oxide was checked by drying it in a drying apparatus at 60 °C until constant weight. The wet graphene oxide was then dispersed in water to obtain 50 ppm dispersion. Graphite with a particle size < 20 µm was used following previous research on the biocidal properties of GO flakes of various particle sizes [44].

The reagents used to prepare phosphate-buffered saline (PBS) included: sodium chloride, potassium chloride and disodium hydrogen phosphate purchased from Sigma-Aldrich (Poznan, Poland). PBS solution with the following composition was prepared: NaCl 8 g/L, KCl 0.2 g/L and Na_2_HPO_4_ 1.44 g/L. A pH of 7.4 was obtained by adding the appropriate amount of 0.1 M NaOH.

Paracetamol Kabi (10 mg/mL) was purchased from Fresenius Kabi Polska sp. z o.o.

### 2.2. Culture Medium

Acetate fermentation of Golden Delicious apples from organic cultivation located in the Silesian Voivodship was carried out. First, a sterilized 1000 mL jar was filled to one-third with apples cut into pieces. The fruit was covered with a solution of water with sucrose (80 g of sucrose dissolved in 1 L of water), which was initially sterilized in an autoclave at 121 °C for 15 min. Finally, the jar was covered with sterile gauze. The contents of the jar were thoroughly mixed 2 times a day. After 2–3 days, it was possible to observe the formation of gas bubbles in the jar, which indicated the alcoholic fermentation reaction in progress, ending when the fruit fell to the bottom of the jar. The solution was then separated from the fruit, poured back into the jar and covered with sterile gauze. From this point on, the process of acetic acid fermentation began and the “mother of vinegar”, which includes BC, live bacteria and yeast, was formed. The fermentation process was completed after 3 weeks. The BC formed in the reaction was gently removed from the surface of the solution, transferred to a sterilized jar and then covered with a small amount of apple cider vinegar. The “mother of vinegar” and apple cider vinegar were stored in the refrigerator.

### 2.3. Production and Purification of BC

For the production of BC, a liquid sucrose medium with a concentration of 110 g/L was prepared and then sterilized in an autoclave at 121 °C for 15 min. A total of 800 mL of liquid sucrose medium, 200 mL of apple cider vinegar obtained earlier and a 1 × 1 cm fragment of “mother of vinegar” were placed in a sterilized 2000 mL laboratory beaker, and then mixed thoroughly. The pH of the medium was also measured and found to be pH = 3.5. Then, the mixture prepared in this way was divided into 4 parts and placed in sterilized 300 mL beakers (Table 1).

Each vessel was covered with a watch glass and then transferred to an incubator preheated to 25 °C. The synthesis of BC was carried out for 12 days by adding appropriate amounts of GO dispersion to the consecutive samples (Table 1). As a result of our previous research on the optimization of GO concentration, a solution with a concentration of 50 ppm was selected. Solutions with lower concentrations of GO at 10 and 25 ppm did not affect the BC synthesis. A 100 ppm solution of GO, on the other hand, stopped the synthesis of BC.

**Figure 1:**
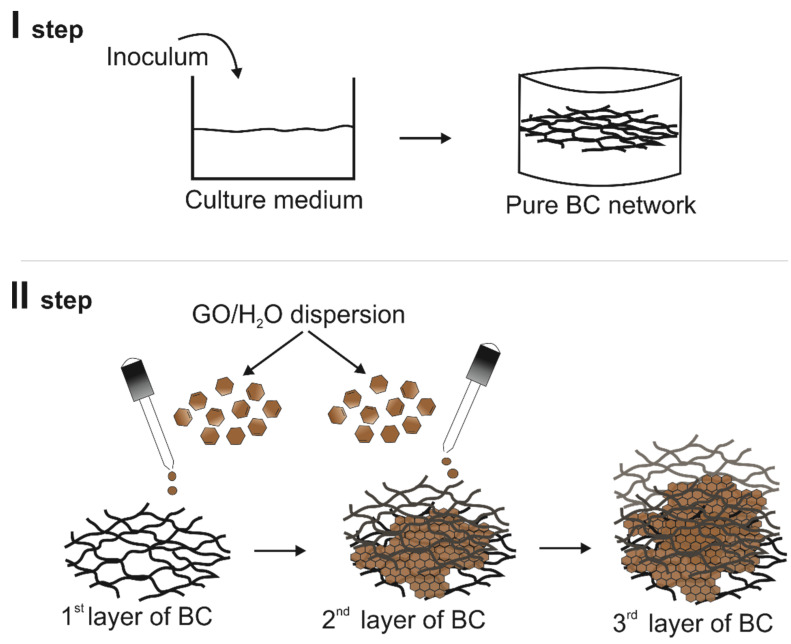
The process of obtaining modified BC.

The stages of this part of the experiment as well as the process of introducing the GO dispersion onto the surface of BC are presented in Figure 1.

After completion of the reaction, membranes from pure BC (BC/0) and from modified BC (BC/GO/I; BC/GO/II; BC/GO/III) were removed from the beakers and rinsed with distilled water to remove residual medium.

**Figure 2:**
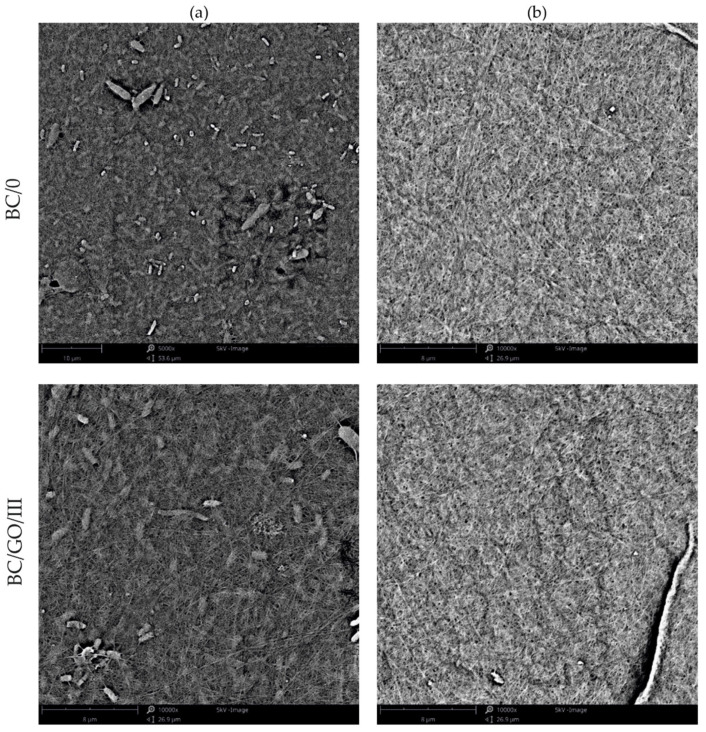
SEM photo of the membrane made from pure BC/0 and sample BC/GO/III: (**a**) after the synthesis process—with visible bacterial cells; (**b**) after KOH purification process.

Then, the process of purifying the membranes of organic residues (bacterial cells) was carried out (Figure 2). For this purpose, each membrane was placed in a separate 300 mL beaker containing 100 mL of 2% *w*/*w* aqueous solution of KOH [30]. The purification process was carried out at 80 °C for 1 h under static conditions. Then, the membranes were washed several times with distilled water until the reaction was neutral.

### 2.4. Thickness Measurement

**Figure 3:**
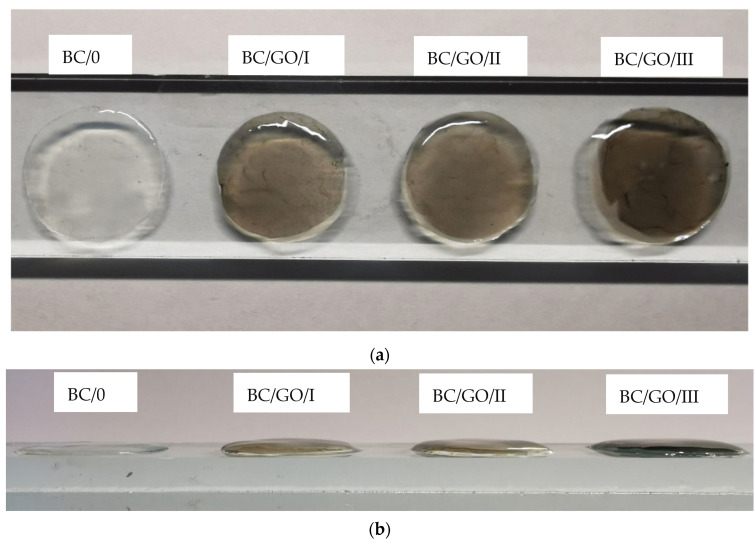
Samples of wet membranes placed on glass, prepared for thickness measurement; (**a**) top view; (**b**) side view.

After the purification and neutralization process of the obtained membranes, their thickness was measured. For this purpose, discs with a diameter of 2 cm were cut from the wet membranes. The membranes were then placed on a glass plate and the thicknesses were read using an electronic micrometer screw (YATO YT-7201). For all obtained membranes, 20 measurements were made (Figure 3), and the obtained results are summarized in Table 2.

In addition to measuring the thickness when wet, the thickness of the dry membranes was also measured. Initially, the membranes were air-dried until a constant weight was obtained. Thickness measurements were made using an electronic thickness gauge (Elmetron MG-411, Zabrze, Poland). In order to avoid spot-crushing of the membrane, a cover glass was used and placed on the membrane at the point where its thickness was measured. Twenty measurements were made for each membrane, and the obtained results are summarized in Table 2.

### 2.5. Measurement of Water Content in Wet Samples and Water Absorption in Dry Samples

The water content was measured for wet and dry membranes. Initially, samples of wet membranes with a diameter of 6 cm were transferred to aluminum dishes with a diameter of 9 cm, and then weighed on a laboratory electronic moisture balance (Radwag MA 110.R, Radom, Poland) with an accuracy of 0.0001 g. The membrane drying process was carried out at 80 °C until a constant mass was obtained.

The discs of membranes dried during the test described above were used to measure the water absorption. The membranes placed on aluminum dishes were soaked in distilled water added in such an amount as to cover their surface and left for 1 h. After this time, the water was removed from the dishes. The excess water was then collected from the membrane using filter paper and the weighing was repeated.

Twenty measurements were made for each wet and dry membrane, and the obtained results are summarized in Table 2.

### 2.6. In Vitro Study of Bioactive Substance Release

The kinetics of the active substance release from the membranes was studied in phosphate-buffered saline (PBS), which mimics the ion concentration, osmolarity and pH of human body fluids. Paracetamol, which is commonly used as a strong analgesic and antipyretic medicine, was selected for the sorption studies of biologically active substances.

At the beginning, a standard curve was prepared. For this purpose, 4 solutions containing PBS (as a solvent) and paracetamol at a concentration of 2.5, 5.0, 10.0 and 20.0 ppm were prepared. The solutions were tested using a UV-Vis spectrophotometer (Thermo Scientific, Evolution 220, Waltham, MA USA), at a wavelength of λ = 243 nm, using PBS as the reference solution.

Then, the sorption and desorption of paracetamol from the membranes obtained in the experiment were studied. Initially, tests were carried out on dry membranes, which, however, proved unsuccessful. Therefore, the experiment was carried out with the use of wet membranes. The membranes in wet form can potentially be used as carriers of an active substance, e.g., a medicine.

In the test, wet BC/0 and BC/GO/I; BC/GO/II; BC/GO/III membranes were placed in separate beakers containing 100 mL of paracetamol solution (at a concentration of 100 ppm) dissolved in PBS. The beakers with the membrane samples were left at room temperature for 24 h. After this time, samples of the solution were taken from the beakers and the absorbance was measured using the UV-Vis spectrophotometer at the wavelength of λ = 243 nm, determining the amount of medicine adsorbed by each tested membrane.

Studies on medicine desorption from membranes followed thereafter. Membranes soaked in paracetamol were transferred to separate beakers, each containing 100 mL of PBS solution, and stirred using a magnetic stirrer at 37 °C. Samples of the solution from these membranes were taken every 10 min and the absorbance values were read. After the measurement, the test solutions were returned to the beakers. The cumulative release factor (C_r_) was determined using the following formula, similar to that calculated in the publication [30]:

(1) $$C_{r}=\left(\frac{C_{t}}{C_{0}}\right)*100\%$$

where C_t_ is concentration released at time t and C_0_ is the concentration of paracetamol in membranes.

### 2.7. Characterization of BC

Membrane surface and cross-section tests were carried out using a high-resolution Phenom ProX scanning electron microscope (SEM) from Thermo Fisher Scientific (Pik Instruments, Poland, Piaseczno), operating at 10 kV. The fiber thicknesses were measured based on the surface images using FiberMetric software developed by PhenomWorld (v. 2.9.0, Eindhoven, The Netherlands). A total of 1000 fiber thickness measurements were taken for each type of membrane. To prepare the cross-sections of the membranes, liquid nitrogen was used, in which the samples were frozen and then broken. The samples were previously coated with a 10 nm gold layer using a Leica EM ACE 200 low-vacuum coater (Vienna, Austria).

The surfaces of the membranes were observed using an OPTA-TECH optical microscope at 10× magnification. The images were taken using the transmission method, in the light passing through the sample.

Wide-angle X-ray scattering (WAXS) studies were performed using URD-65 Seifert diffractometer (Freiberg, Germany) employing the Bragg–Brentano reflection geometry method. CuKα radiation (λ = 1.54 Å) was emitted at an accelerating voltage of 40 kV and an anode current of 30 mA. The monochromatization of the radiation beam was achieved by a graphite monochromatizer placed across the monochromatized beam. A scintillation counter was used as a detector. The tests were carried out in the range of 2θ from 5 to 60° in steps of 0.1°.

Fourier Transform Infrared Spectroscopy (FTIR) measurements were taken using a Nicolet 6700 FT-IR spectrometer (Thermo Electron Corp., Madison, WI, USA) equipped with a photoacoustic MTEC model 300 accessory. The following measurement parameters were used: resolution, 4 cm^−1^; photoacoustic detector; number of scans, 256. For each sample, the spectra were collected over the range of 4000 to 500 cm^−1^. Data collection and post-processing were performed using OMNIC software (v. 9.0, Thermo Electron Corp., Madison, WI, USA).

Thermogravimetric analysis were performed using TA Instruments Q500 Thermogravimetric Analyzer. The measurements were conducted in a nitrogen atmosphere (flow 60 mL/min), in the temperature range of 30 to 500 °C and at a heating rate of 20°/min. TG and DTG curves were analyzed using Universal V2.6D TA Instruments software.

## 3. Results and Discussion

This study describes the possibility of obtaining BC and composites of BC with graphene oxide in situ. The study used microorganisms obtained from Golden Delicious apple vinegar as the literature provides many examples of isolating bacterial strains from fruits and vegetables [5].

The membranes obtained in the experiment, made of pure BC (BC/0) and composite membranes (BC/GO/I, BC/GO/II, BC/GO/III), were treated with KOH. This treatment process is designed to remove organic matter such as bacterial cells. Figure 2a clearly shows the bacterial rods entangled between the BC filaments. On the other hand, in the SEM photo, after the alkali washing process (Figure 2b), the bacterial cells are gone, and only the BC filaments are visible.

**Figure 4:**
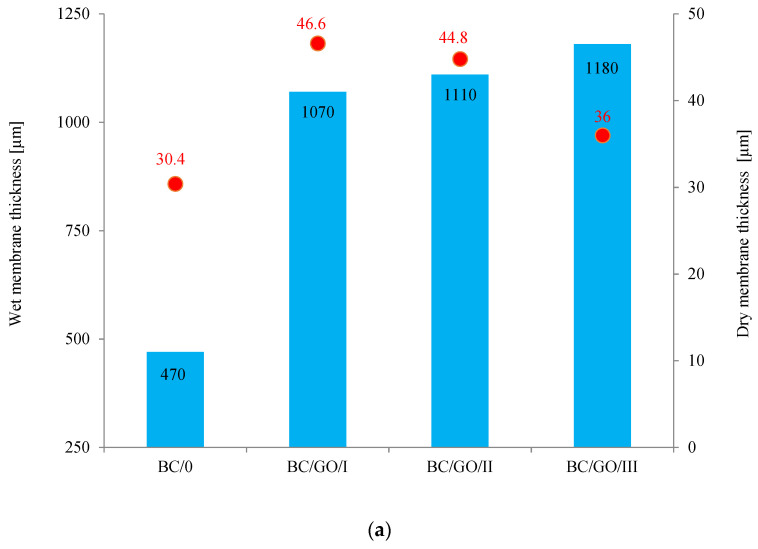

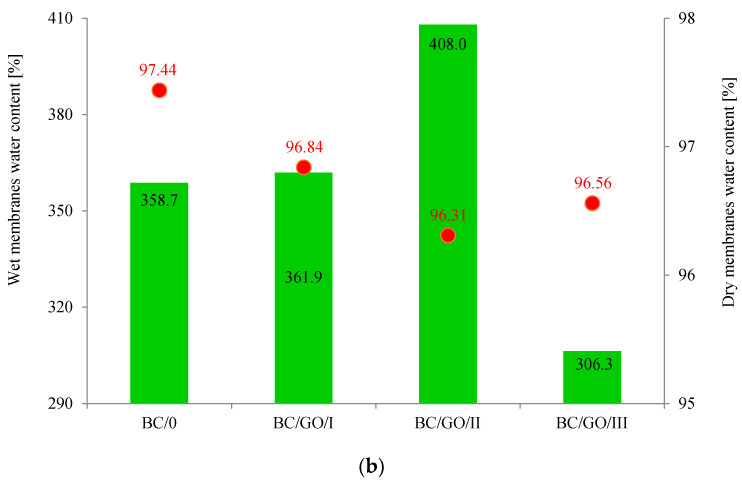
(**a**) Dependence of the thickness of wet and dry membranes on the content of GO in the sample; (**b**) water content of wet membranes and water absorption of dry membranes.

At the beginning of the research, the physicochemical properties of the membranes, such as the thickness and water absorption, were determined for both pure cellulose (BC/0) and composite (BC/GO/I, BC/GO/II, BC/GO/III) membranes. The obtained results are summarized in Figure 4.

As Figure 4a demonstrates, the addition of GO to the growing inoculum significantly increases the thickness of the cellulose composite membranes. For wet membranes, the membrane thickness values increase from 470 µm (BC/0) to 1070, 1110 and 1180 µm for BC/GO/I, BC/GO/II and BC/GO/III membranes, respectively, which is an increase of over 130%. Thus, the addition of GO promotes an increase in the amount of BC produced or the thickness of the nanofibers formed. In addition, it is observed that an increase in the GO contents in the sample (3.7; 5.4; 7.1% of GO) results in a slight increase in the thickness of the composite membrane samples. However, after drying of all tested membranes, their thickness drops drastically to 30.4 µm for BC/0. It is closely related to the formation of inter- and intramolecular hydrogen bonds during drying of cellulose [45,46]. Moreover, an increase in the amount of GO added to the BC/GO/III sample in the amount of up to 7.1% *w*/*w* probably causes the formation of additional hydrogen bonds, which have a direct impact on the formation of a more compact structure, resulting in a decrease in the thickness of this membrane.

Studies have also shown that the water content of wet membranes ranges from 306.3 to 408.0% (Figure 4b). For pure BC membranes, the water content is ~359%. The addition of GO in the amount of 5.4% *w*/*w* causes an approx. 14% increase in the water content in the BC/GO/II membrane. The result obtained for the BC/GO/III wet membrane may suggest that the presence of GO caused the formation of additional hydrogen bonds.

The water absorption tests carried out for dry membranes (Figure 4b) demonstrated that this property is practically independent of the type of membrane; it ranges from approx. 96% to approx. 97% for the BC/0 membrane. Lower water absorption values for dry membranes BC/GO/I, BC/GO/II and BC/GO/III may also prove the formation of additional hydrogen bonds between BC and GO [36].

**Figure 5:**
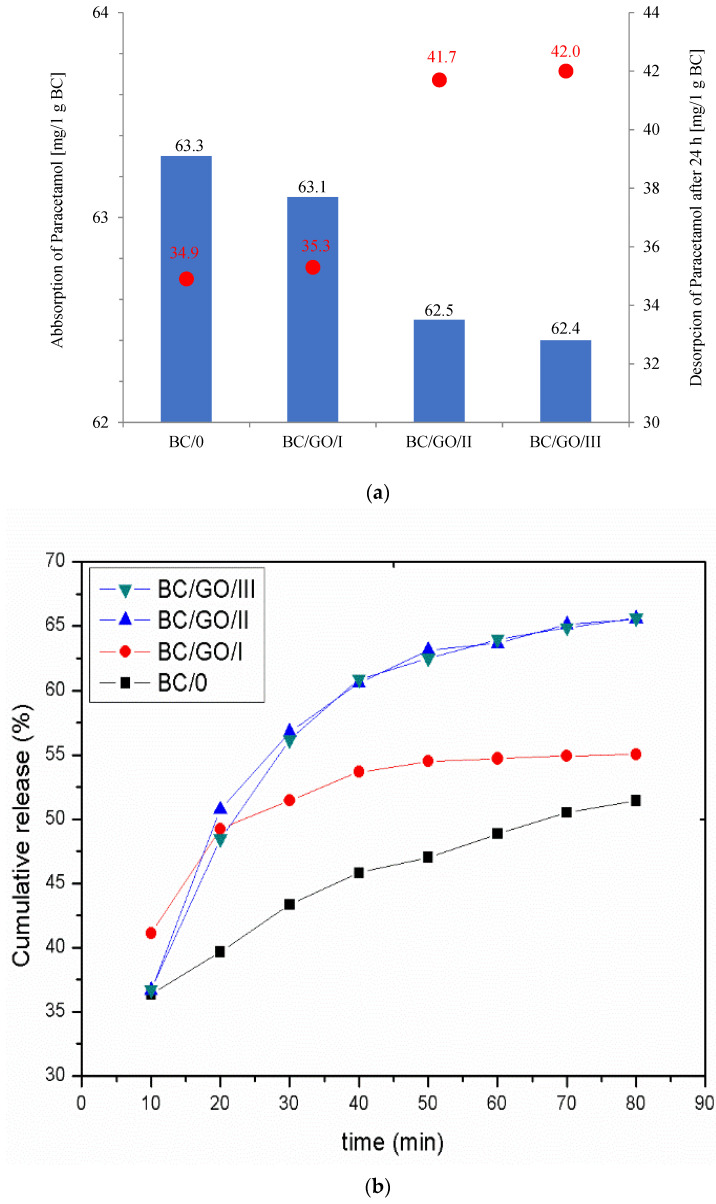
(**a**) Graph of active substance absorption on membranes and active substance desorption from membranes after 24 h; (**b**) Paracetamol release from pure cellulose (BC/0) and composite (BC/GO/I, BC/GO/II, BC/GO/III) membranes to PBS solution.

Analyzing the sorption properties (Figure 5a) of the obtained membranes against paracetamol, it can be concluded that the medicine is well-sorbed on the membranes obtained in the experiment. The amount of adsorbed medicine is 63.3 mg/g BC (for the BC/0 membrane), and this slightly decreases with the increase in GO content in composite membranes to the value of 62.4 mg/g BC (for the BC/GO/III membrane). The desorption tests, on the other hand, showed that the composite membranes desorbed more paracetamol than the BC/0 sample (34.9 mg/g BC), in the following order: BC/GO/III (42.0 mg/g BC), BC/GO/II (41.7 mg/g BC) and BC/GO/I (35.3 mg/g BC).

Interesting results were obtained during the study of the kinetics of the medicine release from the tested membranes (Figure 5b). They show that the BC/0 sample desorbs paracetamol slowly but steadily; however, the release rate is less than 55%. On the other hand, the membrane with the lowest GO content (3.7% *w*/*w*) is characterized by a more intense medicine desorption in the initial phase of the release, which slows down after 40 min and remains at the level of ~55%. The medicine substance release curves for the BC/GO/III and BC/GO/II composite membranes have similar characteristics, and their release rates after 24 h are high, amounting to ~67%. The results obtained in the study of the release of paracetamol from BC-based composite membranes with the addition of GO reach values that are much higher than those described by the team of Urbin et al. [30].

The examination of the physicochemical properties of the membranes was supplemented with microscopic examinations. The use of scanning electron microscopy allowed for the analysis of the surface of the obtained membranes, their cross-sections and the thickness of cellulose nanofibers that make up the membrane structure.

**Figure 6:**
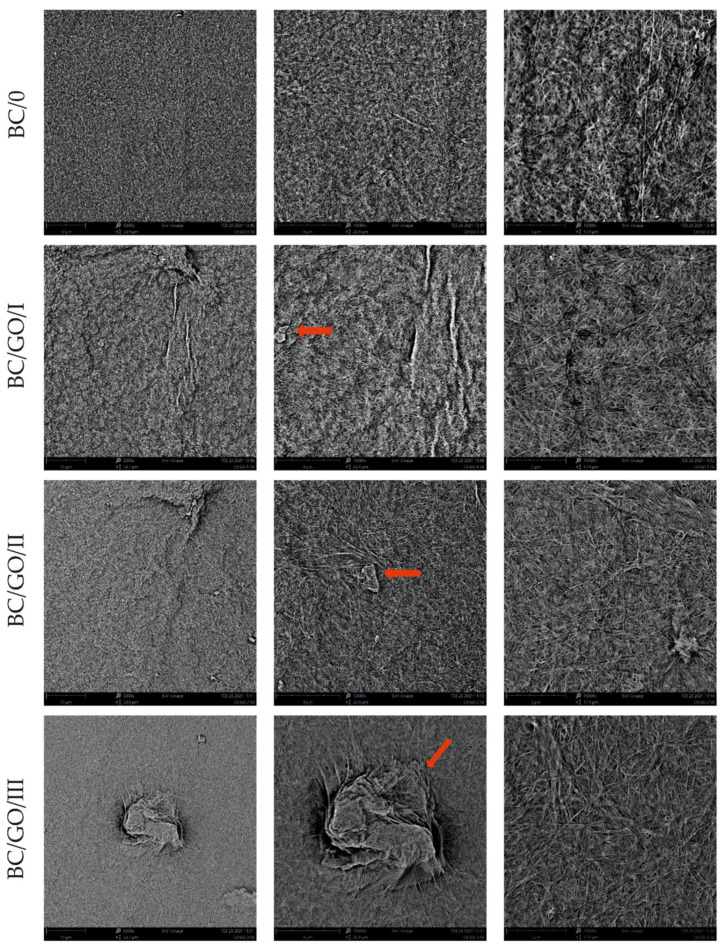
SEM pictures of the surface of BC (BC/0) samples and BC/GO/I, BC/GO/II, BC/GO/III composites at a magnification of 5000×, 10,000×, 15,000×.

The SEM photos (Figure 6) clearly show that the membranes are made of BC nanofibers, the arrangement of which on the surface is completely random, as in the classic non-woven fabric. Additionally, for the membranes BC/GO/I, BC/GO/II and BC/GO/III, the presence of inclusions (red arrow), which come from the GO addition, can be observed. SEM studies have shown that the graphene oxide flakes do not appear directly on the surface of composite membranes but are covered with the web of BC nanofibers.

**Figure 7:**
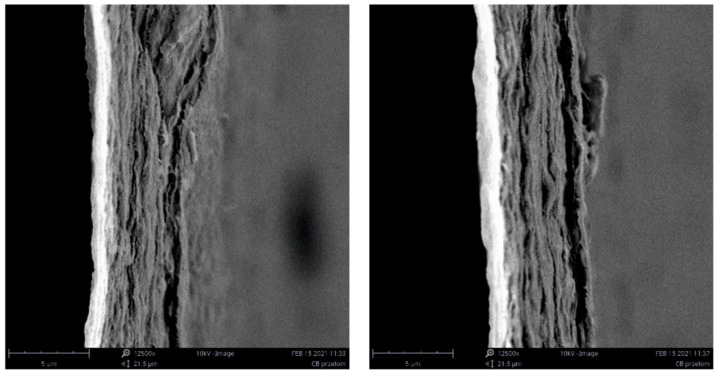
SEM photos of the BC/0 membrane fracture.

Scanning electron microscopy also allowed observing fractures of the obtained membranes. The sample photos show a pure BC membrane (Figure 7). In Figure 7 of the BC/0 membrane, we can see that the membrane is made of nanofibers that are arranged in layers, parallel to each other.

The use of FiberMetric software from PhenomWorld (v. 2.9.0, Eindhoven, The Netherlands) for the analysis of SEM images of the membranes allowed performing thickness measurements, which are summarized in Table 3.

Measurements of the thickness of the BC fibers forming the membranes (Table 3) demonstrated that the thinnest fibers with a diameter of 85 ± 91 nm were characteristic of the BC/0 membrane, which is consistent with the literature reports [47]. The thickness of the composite fibers, on the other hand, was slightly larger and ranged from 96 to 100 nm for subsequent membranes. Therefore, it can be concluded that the addition of GO strongly influences the formation process of nanofibrils and cellulose fibrils, disrupting the process of their formation, which confirms the results described in the literature [32]. Moreover, in the histograms (Table 3), pure BC fibers can be observed to have a low thickness spread, unlike the composite membranes.

The presence of GO in the inoculum results in a slight increase in the thickness of the BC fibers that form composite membranes. The thickness of the fibers seems to be related to the thickness of the membranes themselves (Table 2) for both wet and dry membranes. Thicker cellulose fibers form thicker composite membranes (BC/GO/I, BC/GO/II, BC/GO/III). In the case of wet membranes, the thicker BC fibers form a loose structure that contains a lot of water in the inter-fiber spaces. However, after drying the composite, membrane thickness is still 20 to 56% greater than that of the BC/0 membranes. However, a decrease in the thickness of dry membranes is observed as the GO content of the composite increases. This may be related to the amount of hydrogen bonding between cellulose particles and graphene oxide, which results in a very dense membrane.

**Figure 8:**
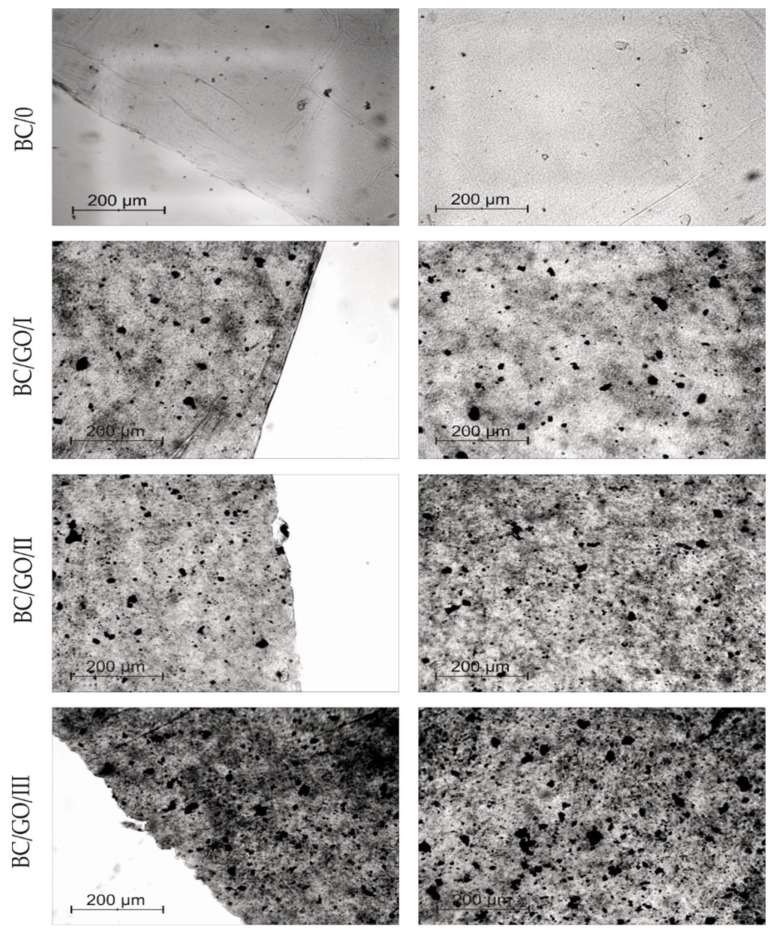
Optical microscope photos of BC (BC-0) samples and BC/GO/I, BC/GO/II, BC/GO/III composites.

Optical microscopy allows observing the structure of BC membranes and the degree of dispersion of the GO additive in the BC/GO/I, BC/GO/II and BC/GO/III membranes (Figure 8).

Figure 8 clearly shows the effect of the amount of GO on the internal structure of the obtained membranes. Membrane BC/0 is practically transparent, with the exception of small, foreign impurities on its surface. On the other hand, the photographs of the BC/GO/I, BC/GO/II and BC/GO/III membranes show clear GO inclusions present in the transparent structure of the dry BC. The GO additive is not uniformly dispersed. This may indicate that GO introduced into the reaction was woven into the network of BC nanofibers randomly. Moreover, it should be noted that in the photographs of the composite membranes (Figure 8), no agglomeration of the GO additive is observed. They only show an increase in the number of GO flakes in the direction from the BC/GO/I membrane, through BC/GO/II membrane to BC/GO/III membrane, in line with the amount of GO addition. We know from our own experience that the aqueous dispersion of GO in solutions with an acidic pH, which in our case is 3.5, does not tend to form agglomerates.

**Figure 9:**
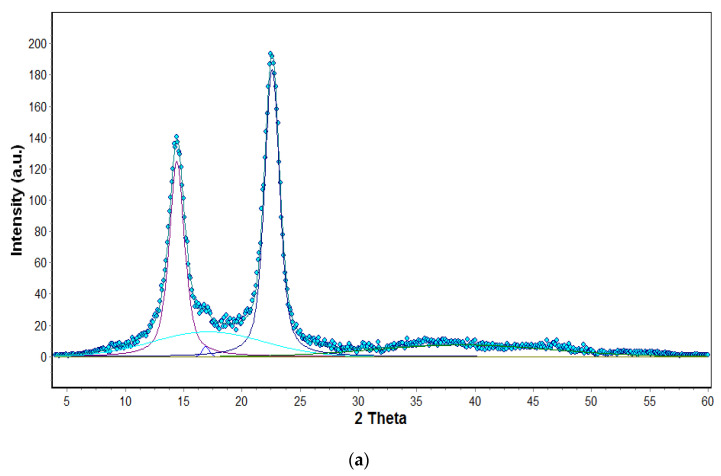

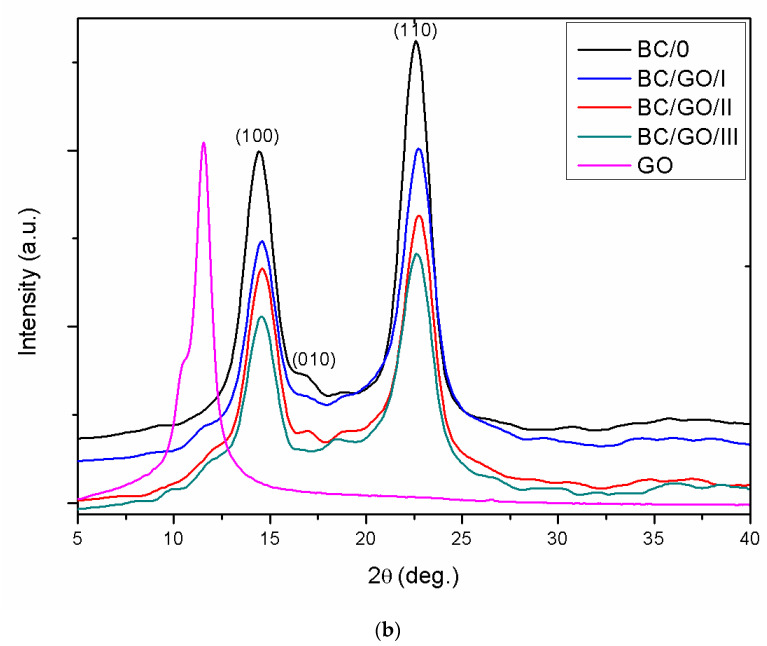
(**a**) Distribution of XRD diffraction patterns into crystalline and amorphous components; (**b**) Summary of diffractograms of pure BC (BC/0) and composites (BC/GO/I, BC/GO/II, BC/GO/III) as well as pure GO membranes.

The research on the structure of the obtained membranes was extended by the WAXS study. The structural parameters of the obtained cellulose membranes were determined on the basis of WAXS diffraction curves. The crystallinity index (CI) of obtained materials was determined on the basis of WAXS analysis by the peak deconvolution method [48]. For this purpose, each WAXS pattern was distributed into individual crystalline and amorphous components using the WaxsFit software [49]. This software was also used to calculate the crystallinity index. In the software, deconvolution is performed by means of an approximation method. It consists of the construction of a theoretical curve, which is composed of functions related to individual crystalline peaks and amorphous maxima. The shape of each peak was approximated using a linear combination of the Gaussian and Cauchy’s functions. The parameters of these functions are found through best fitting of the theoretical curve to the experimental one using a suitable optimization procedure. The theoretical curve was fitted to the experimental data using Rosenbrock’s double-criteria optimization method, which is described by Rabiej et al. [50]. The crystallinity index was determined as the ratio of the sum of the surface areas under the crystalline peaks to the total area under the scattering curve. An example of the distribution of BC XRD diffraction pattern into crystalline and amorphous components with the use of this software is shown in Figure 9a.

Figure 9b shows the WAXS diffraction patterns of pure cellulose (BC/0) membranes, composite membranes (BC/GO/, BC/GO/II, BC/GO/III) and GO. The curves show the characteristic peaks for type I cellulose, whose angular positions fall at 2 Theta of 14.5°, 16.7° and 22.7° and correspond to the planes (100), (010) and (110), respectively [51,52]. This means that the addition of GO did not change the structure of the BC, as no significant shifts were observed. The WAXS curve for the GO, in turn, shows one characteristic high intensity peak at 2 Theta of 11°. The intensity of the 14.5° peak is much higher than that at 16.7°, which results from the typical features of BC [53]. Analyzing the shape of the curves for composite membranes (BC/GO/I, BC/GO/II, BC/GO/III), one can see slight fluctuations within the 2 Theta scattering angle of 11º, which are not present in the case of pure cellulose (BC/0). This is undoubtedly the effect of the presence of GO in the cellulose matrix of these membranes.

The conducted research shows that the degree of crystallinity of pure BC (BC/0) is high and amounts to 66%. This is a typical value for BC, a polymer of high chemical purity [41]. Analysis of the results also indicates a decrease in the degree of crystallinity with an increase in the GO content in the cellulose matrix. GO addition in the amount of 3.7, 5.4 and 7.1% *w*/*w* reduces the degree of crystallinity to 51, 47 and 40% for BC/GO/I, BC/GO/II and BC/GO/III samples, respectively. Dhar et al. in their work described a similar correlation but with reduced graphene oxide, whereby a higher concentration of rGO decreased the crystallinity index up to 5% [41]. This phenomenon is explained by the fact that additives, and especially nanoadditives, disturb the formation process of BC nanofibrils, so that there is a minority of highly ordered crystalline areas in the overall structure of the material [54]. Troncoso et al. in their work described in detail how graphene nanoadditives can interfere with the formation of BC nanofibrils, thus reducing the degree of crystallinity [32].

**Figure 10:**
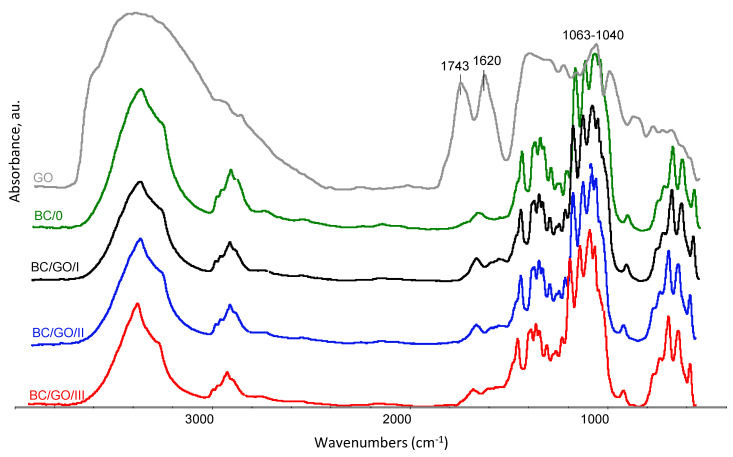
Summary of FTIR spectra for the following samples: pure BC (BC/0), BC/GO/I, BC/GO/II and BC/GO/III composite membranes and GO. The spectra were recorded in the range of 4000 ÷ 400 cm^−1^.

In order to investigate the interactions between BC and the GO additive, FTIR spectroscopy studies were performed. Figure 10 summarizes the FTIR curves for the tested membranes.

The comparison of FTIR spectra (Figure 10) shows that the samples of BC with the GO addition (BC/GO/I, BC/GO/II, BC/GO/III) retained all the characteristic features of the pure BC bands, which confirms the behavior of this chemical structure. In all the obtained spectra, a characteristic broad absorption band of the O-H group stretching vibrations appears at wave numbers in the range of approximately 3600 ÷ 3240 cm^−1^. There is also a slight decrease in intensity and bandwidth at a maximum of 3350 cm^−1^ for the stretching vibrations of the O-H oscillators along with the amount of GO added to BC. This may indicate the formation of alkali cellulose as a result of its treatment with potassium hydroxide. The spectra show characteristic vibration bands of the C-H group in cellulose I and cellulose II, at 2895 cm^−1^ and 1334 cm^−1^ [54]. The presence of these bands proves that the introduction of subsequent portions of GO during the synthesis has no effect on this group. There are no significant changes in the wavenumber range: 1162 cm^−1^, 1113 cm^−1^, 1063 cm^−1^ or 1040 cm^−1^ of the C–O–C group, occurring in the glucopyranose ring and ether bridges [55]. Additionally, the effects of GO on skeletal oscillators, resulting in bands at the wavenumbers around 896 cm^−1^, are not observed. In Figure 10, a slight increase in the intensity of the bands can also be observed at 1652 cm^−1^ and 1576 cm^−1^ for the samples of composite membranes BC/GO/I, BC/GO/II and BC/GO/III. These peaks correspond to the C=C double bond vibrations in ring structures, which are one of the main bands characteristic of GO [56,57].

**Figure 11:**
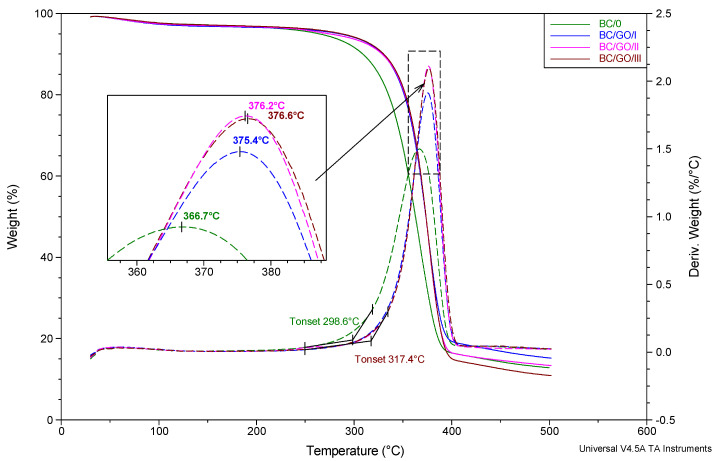
Summary of TG and dTG curves for membrane BC/0, BC/GO/I, BC/GO/II, BC/GO/III samples. Heating rate 20°/min. N_2_ atmosphere flow 60 mL/min.

Based on the analysis of the thermogravimetric study results (Figure 11), it can be concluded that the process of thermal dissociation of the BC matrix of the tested membranes takes place in a single stage and in a fairly wide temperature range: from approx. 250 to 400 °C. The temperature of the extrapolated beginning of the matrix degradation process, determined on the basis of DTG curves, is the lowest for the BC/0 membrane (298.6 °C) and the highest for the BC/GO/III membrane (317.4 °C). The temperature of the maximum thermal decomposition rate of membranes in nitrogen atmosphere, expressed as the location of the respective maxima on the DTG curves is also the lowest for the sample without the GO addition (366.7 °C) and increases monotonically as the modifier content increases up to 376.6 °C for BC/GO/III membrane. It should be noted, however, that the characteristic thermal decomposition temperatures, for the membranes modified with GO, vary only slightly as a function of GO content.

## 4. Conclusions

A simple method of obtaining composite membranes from BC, containing the addition of GO (from 3.7 to 5.1% *w*/*w*), was developed. The in situ bioreaction method ensured the optimal pH of the solution (pH = 3.5), preventing GO agglomeration, and optical microscopy confirmed the good dispersibility of the nanoadditive in the BC matrix. The possibility of using the obtained composite membranes as a potential active dressing with sustained-release analgesic medicine—paracetamol—was also investigated. The BC/GO wet composite membranes selected for these tests (~400% water content in the membrane, thickness of ~1100 µm, BC nanofiber diameter of ~100 nm) were characterized by a slow desorption time, constant drug concentration over time and a high drug release rate after 24 h (~80%). The morphology of the surface and cross-sections of the obtained membranes, examined using SEM, did not show any significant differences in their structure, but revealed the presence of GO flakes woven into the structure of BC nanofibers. The SEM analysis and the study of the degree of crystallinity (WAXS) confirmed the effect of the GO addition on the large dispersion of the BC nanofiber diameter and the related decrease in crystallinity from 66% (for the BC/0 sample) to 40% (for the BC/GO/III sample). Infrared spectroscopy (FTIR), X-ray structure studies (WAXS) as well as thermal analysis (TGA) demonstrated the presence of GO in the BC matrix and interactions between the matrix and the additive. The shift of the characteristic temperatures of the BC thermal degradation process, with the temperature of the highest process rate —in the extreme case (BC/0 membrane) by less than 10 °C and the temperature of the extrapolated onset of transformation—being up to nearly 19 °C, clearly demonstrates the increase in thermal stability of the tested membranes influenced by GO.

## Figures and Tables

**Table 1:** Designation of samples and their composition.

Sample Designation	BC/0	BC/GO/I	BC/GO/II	BC/GO/III
Incubation time [h]/[days]	Amount of GO added [mL] (GO dispersion with a concentration of 50 ppm)			
96/4	-	10	10	10
144/6	-	10	10	10
192/8	-	-	10	10
240/10	-	-	-	10
BC content [% *w*/*w*]	100	96.3	94.6	92.9
GO content [% *w*/*w*]	0	3.7	5.4	7.1

**Table 2:** Summary of the results of thickness and water absorption measurements for wet and dry membranes.

Sample Designation	BC/0	BC/GO/I	BC/GO/II	BC/GO/III
Thickness of wet membranes [µm]	470.0 ± 8.1	1070.0 ± 74.8	1110.1 ± 37.4	1180.0 ± 24.5
Thickness of dry membranes [µm]	30.4 ± 7.1	46.6 ± 7.9	44.8 ± 12.7	36.0 ± 12.8
Water content in wet membranes [%]	358.7 ± 45.0	361.9 ± 43.2	408.0 ± 48.4	306.3 ± 37.9
Water absorption of dry membranes [%]	97.4 ± 5.1	96.8 ± 4.5	96.3 ± 3.9	96.6 ± 4.0

**Table 3:** Thickness dispersion of BC fibers in the following membranes: BC/0, BC/GO/I, BC/GO/II and BC/GO/III, measured using FiberMetric software: (a) photo of the sample with measurements (at 10,000× magnification); (b) a histogram of BC fibers diameter distribution; (c) mean fiber diameter (nm) measured in the test range from 0 to 100 nm.

	(a)	(b)	(c)
BC/0	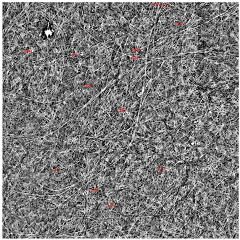	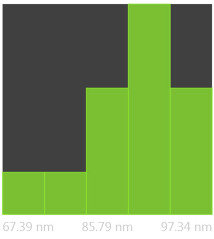	85 ÷ 91 nm
BC/GO/I	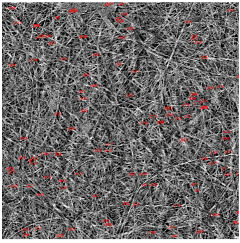	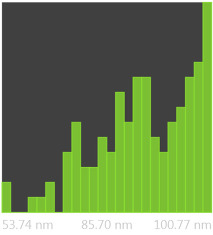	98 ÷ 100 nm
BC/GO/II	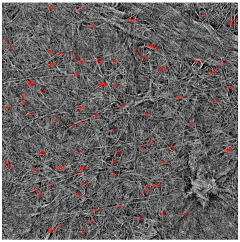	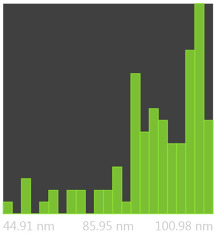	96 ÷ 98 nm
BC/GO/III	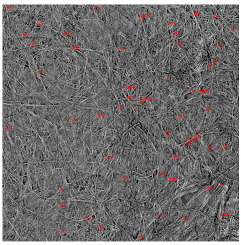	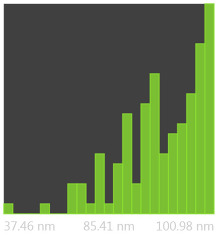	97 ÷ 100 nm

## Data Availability

Not applicable.
